# Brain Aging in Specific Phobia: An ENIGMA‐Anxiety Mega‐Analysis

**DOI:** 10.1002/hbm.70595

**Published:** 2026-07-06

**Authors:** Kimberly V. Blake, Kevin Hilbert, Jonathan C. Ipser, Laura K. M. Han, Janna Marie Bas‐Hoogendam, Fredrik Åhs, Jochen Bauer, Katja Beesdo‐Baum, Johannes Björkstrand, Laura Blanco‐Hinojo, Joscha Böhnlein, Robin Bülow, Marta Cano, Narcis Cardoner, Xavier Caseras, Udo Dannlowski, Mats Fredrikson, Liesbet Goossens, Hans J. Grabe, Dominik Grotegerd, Tim Hahn, Alfons Hamm, Ingmar Heinig, Martin J. Herrmann, David Hofmann, Hamidreza Jamalabadi, Andreas Jansen, Merel Kindt, Tilo Kircher, Anna L. Klahn, Katja Koelkebeck, Axel Krug, Elisabeth J. Leehr, Martin Lotze, Juergen Margraf, Markus Muehlhan, Igor Nenadić, Wenceslao Peñate, Andre Pittig, Jens Plag, Jesús Pujol, Jan Richter, Isabelle C. Ridderbusch, Francisco Rivero, Axel Schäfer, Judith Schäfer, Anne Schienle, Elisabeth Schrammen, Koen Schruers, Esther Seidl, Rudolf M. Stark, Benjamin Straube, Thomas Straube, Andreas Ströhle, Boris Suchan, Lea Teutenberg, Sophia I. Thomopoulos, Carlos Ventura‐Bort, Renee M. Visser, Henry Völzke, Albert Wabnegger, André Wannemüller, Julia Wendt, Hans‐Ulrich Wittchen, Katharina Wittfeld, Yunbo Yang, Anna Zilverstand, Peter Zwanzger, Lianne Schmaal, Moji Aghajani, Daniel S. Pine, Paul M. Thompson, Nic J. A. van der Wee, Dan J. Stein, Ulrike Lueken, Nynke A. Groenewold

**Affiliations:** ^1^ Department of Psychiatry and Mental Health, Neuroscience Institute University of Cape Town Cape Town South Africa; ^2^ Department of Psychology HMU Health and Medical University Erfurt Erfurt Germany; ^3^ Centre for Youth Mental Health University of Melbourne, Orygen Parkville Victoria Australia; ^4^ Department of Developmental and Educational Psychology Leiden University Leiden the Netherlands; ^5^ Department of Psychiatry Leiden University Medical Center Leiden the Netherlands; ^6^ Leiden Institute for Brain and Cognition Leiden the Netherlands; ^7^ Department of Psychology and Social Work Mid Sweden University Östersund Sweden; ^8^ University Clinic for Radiology University of Münster Münster Germany; ^9^ Behavioral Epidemiology, Institute of Clinical Psychology and Psychotherapy Dresden University of Technology (TUD) Dresden Germany; ^10^ Department of Psychology Lund University Lund Sweden; ^11^ MRI Research Unit, Department of Radiology Hospital del Mar Barcelona Spain; ^12^ Institute for Translational Psychiatry University of Münster Münster Germany; ^13^ Department for Clinical Psychology and Translational Psychotherapy University of Münster Münster Germany; ^14^ Institute of Diagnostic Radiology and Neuroradiology University Medicine Greifswald Greifswald Germany; ^15^ Sant Pau Mental Health Research Group Barcelona Spain; ^16^ Centre for Neuropsychiatric Genetics and Genomics Cardiff University Cardiff UK; ^17^ Department of Psychiatry Medical School and University Medical Center OWL, Protestant Hospital of the Bethel Foundation, Bielefeld University Bielefeld Germany; ^18^ German Center for Mental Health (DZPG), site Jena Magdeburg Germany; ^19^ Center for Intervention and Research on Adaptive and Maladaptive Brain Circuits Underlying Mental Health (C‐I‐R‐C), site Jena Magdeburg Germany; ^20^ Department of Psychology Uppsala University Uppsala Sweden; ^21^ Department of Psychiatry and Neuropsychology Maastricht University Medical Center Maastricht the Netherlands; ^22^ Department of Psychiatry and Psychotherapy University Medicine Greifswald Greifswald Germany; ^23^ Institute of Psychology University of Greifswald Greifswald Germany; ^24^ Institute of Clinical Psychology and Psychotherapy Technische Universität Dresden Germany; ^25^ Department of Psychiatry, Psychosomatics and Psychotherapy University Hospital Würzburg Würzburg Germany; ^26^ Institute of Medical Psychology and Systems Neuroscience University of Münster Münster Germany; ^27^ Department of Psychiatry and Psychotherapy University of Marburg Marburg Germany; ^28^ Center for Mind, Brain and Behavior (CMBB), Marburg Marburg Germany; ^29^ University of Amsterdam Amsterdam the Netherlands; ^30^ Department of Psychiatry and Neurochemistry, Institute of Neuroscience and Physiology, Sahlgrenska Academy University of Gothenburg Gothenburg Sweden; ^31^ Medical School and University Medical Center OWL, Protestant Hospital of the Bethel Foundation, Department of Psychiatry and Psychotherapy Bielefeld University Bielefeld Germany; ^32^ Department of Psychiatry and Psychotherapy University Hospital Bonn Bonn Germany; ^33^ Functional Imaging Unit, Institute of Diagnostic Radiology and Neuroradiology University Medicine Greifswald Greifswald Germany; ^34^ Mental Health Research and Treatment Center Ruhr‐Universitaet Bochum Germany; ^35^ Department of Psychology, Faculty of Human Sciences MSH Medical School Hamburg Hamburg Germany; ^36^ ICAN Institute of Cognitive and Affective Neuroscience MSH Medical School Hamburg Hamburg Germany; ^37^ Department of Clinical Psychology, Psychobiology and Methodology University of La Laguna La Laguna Spain; ^38^ Translational Psychotherapy, Institute of Psychology University of Göttingen Göttingen Germany; ^39^ Faculty of Medicine, Institute for Mental Health and Behavioral Medicine HMU Health and Medical University Potsdam Potsdam Germany; ^40^ Department of Experimental Psychopathology, Institute for Psychology Hildesheim University Hildesheim Germany; ^41^ Universidad Europea de Canarias La Orotava Spain; ^42^ Bender Institute of Neuroimaging Justus Liebig University Giessen Giessen Germany; ^43^ Center for Mind, Brain and Behavior Philipps‐University Marburg Marburg Germany; ^44^ University of Graz Austria; ^45^ SBA Research Vienna Austria; ^46^ Department of Psychotherapy and Systems Neuroscience Justus Liebig University Giessen Giessen Germany; ^47^ Department of Psychiatry and Psychotherapy Philipps‐University Marburg Marburg Germany; ^48^ Department of Psychiatry and Psychotherapy, Campus Charité Mitte Charité ‐ Universitätsmedizin Berlin Berlin Germany; ^49^ Neuropsychological Therapy Centre, Faculty of Psychology Ruhr University Bochum Bochum Germany; ^50^ Imaging Genetics Center, Mark and Mary Stevens Neuroimaging and Informatics Institute, Keck School of Medicine University of Southern California Los Angeles California USA; ^51^ Department of Psychology University of Potsdam Potsdam Germany; ^52^ Institute for Community Medicine University Medicine Greifswald Greifswald Germany; ^53^ Department of Biological Psychology and Affective Science, Faculty of Human Sciences University of Potsdam Potsdam Germany; ^54^ Department of Psychiatry Ludwig‐Maximilians‐University Munich Germany; ^55^ Department of Psychiatry & Behavioral Sciences University of Minnesota Minneapolis Minnesota USA; ^56^ Department of Psychiatry and Psychotherapy Ludwig‐Maximilian University of Munich Munich Germany; ^57^ Institute of Education & Child Studies, Section Forensic Family & Youth Care Leiden University Leiden the Netherlands; ^58^ Emotion and Development Branch, National Institute of Mental Health Bethesda Maryland USA; ^59^ SA‐MRC Unit on Risk and Resilience in Mental Disorders University of Cape Town Cape Town South Africa; ^60^ Department of Psychology Humboldt‐Universität zu Berlin Germany; ^61^ German Center for Mental Health (DZPG), partner site Berlin/Potsdam Berlin and Potsdam Germany

**Keywords:** brain age, brain morphometry, machine learning, mega‐analysis, neuroimaging, phobic disorders

## Abstract

Specific phobia (SPH) is a prevalent anxiety disorder and may involve advanced biological aging. However, limited brain age research has been conducted in anxiety disorders. This mega‐analysis investigated brain aging in SPH participants within the ENIGMA‐Anxiety Working Group. 3D brain structural MRI scans from 17 international samples (600 SPH individuals, of whom 504 formally diagnosed and 96 questionnaire‐based cases; 1134 controls; age range: 22–75 years) were processed with FreeSurfer. Brain age was estimated from 77 subcortical and cortical regions with a publicly available ENIGMA brain age model. The brain‐predicted age difference (brain‐PAD) was calculated as brain age minus chronological age. Linear mixed‐effects models examined group differences in brain‐PAD and moderation by age. No significant group difference in brain‐PAD manifested (*β*_diagnosis_ [SE] = 0.37 years [0.43], *p* = 0.39). A negative diagnosis‐by‐age interaction was identified, which was most pronounced in formally diagnosed SPH (*β*_diagnosis‐by‐age_ = −0.08 [0.03], pFDR = 0.02). This interaction remained significant when excluding participants with anxiety comorbidities, depressive comorbidities, and medication use. Post hoc analyses revealed a group difference for formal SPH diagnosis in younger participants (22–35 years; *β*_diagnosis_ = 1.20 [0.60], *p* < 0.05, mixed‐effects *d* [95% confidence interval] = 0.14 [0.00–0.28]), but not older participants (36–75 years; *β*_diagnosis_ = 0.07 [0.65], *p* = 0.91). Brain aging did not relate to SPH in the full sample. However, a diagnosis‐by‐age interaction was observed across analyses, and was strongest in formally diagnosed SPH. Post hoc analyses showed subtle advanced brain aging in young adults with formally diagnosed SPH. Taken together, these findings indicate the importance of clinical severity, impairment, and persistence, and may suggest a slightly earlier end to maturational processes or subtle decline of brain structure in SPH.

## Three Key Points

Question: Do adults with specific phobia (SPH) demonstrate greater relative brain age compared to healthy controls (HC)?

Finding: This ENIGMA‐Anxiety mega‐analysis did not detect advanced brain aging in adult SPH participants compared to HCs. However, a robust diagnosis‐by‐age interaction was observed, with post hoc analyses suggesting slightly older appearing brains in young adults with formally diagnosed SPH.

Meaning: These findings may provide preliminary evidence for a slight acceleration of brain maturational processes or subtle decline of brain structure in young adults with SPH.

## Graphical abstract

This ENIGMA‐Anxiety mega‐analysis did not find advanced brain aging across a wide age range in adult participants with specific phobia, compared to controls. However, a robust diagnosis‐by‐age interaction was observed, with greater relative brain age in young adults with formally diagnosed specific phobia.

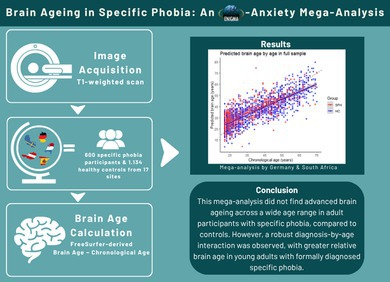

## 1 Introduction

Specific phobia (SPH) involves fear or anxiety about a specific object or situation that is out of proportion to the actual threat posed (American Psychiatric Association 2022). SPH is the most prevalent anxiety disorder, usually developing during childhood, with a lifetime prevalence of 2.6%–12.5% across countries (Kessler et al. 2012; Wittchen et al. 2011). Moreover, subclinical fears which have the potential to develop into clinical SPH are a common phenomenon (Wardenaar et al. 2017). SPH may predict risk for other anxiety or depressive disorders, particularly during the first two decades of life (Boehnlein et al. 2020; De Vries et al. 2019; Lieb et al. 2016; Trumpf et al. 2010). Neurobiological research is needed to unravel the biological correlates of aging in SPH, as anxiety disorders are associated with increased risk of early mortality (Meier et al. 2016). Further, anxiety disorders relate to physiological signs of aging, such as increased oxidative stress, and potential neural markers of aging, such as decreased white matter (WM) and gray matter (GM) density (Perna et al. 2016). The current study addresses this need by examining relations between SPH and brain aging.

Brain age models inform our understanding of brain health and aging. Such models are generally trained on large datasets of healthy controls (HCs) to learn neurostructural correlates of chronological age (Franke et al. 2010; Franke and Gaser 2019). These models are subsequently applied to unseen datasets to estimate brain age at the individual level. Each participant's chronological age is subtracted from their brain age, producing the brain predicted age difference (brain‐PAD). When an individual's brain is estimated to be older than their chronological age (reflected in a positive brain‐PAD), this may indicate underlying health issues, such as mental illness (Cole et al. 2018; Dörfel et al. 2023). Further, brain age has been associated with an increased risk of all‐cause mortality (Cole et al. 2018). Thus, brain age models may identify unhealthy aging patterns, demonstrating potential to identify risk for psychopathology during aging (Cole et al. 2018, 2017).

Most brain age research in psychiatry has so far examined psychotic and mood disorders, with studies finding advanced brain aging in these groups compared to HCs (Blake et al. 2023; Ballester et al. 2022; Han et al. 2020; Kaufmann et al. 2019), and a positive association between brain‐PAD and symptom severity (Han et al. 2021; Koutsouleris et al. 2014; Lieslehto et al. 2021; McWhinney et al. 2021). Further, studies found a potential neuroprotective effect of psychotropic medications (Han et al. 2021; Van Gestel et al. 2019). Evidence therefore indicates that psychopathology is associated with advanced structural brain aging; however, research within anxiety disorders, the most common group of psychiatric disorders with the earliest age of onset, remains limited.

To the best of our knowledge, only one study has considered brain‐PAD in a sample of adults (18–57 years) with anxiety disorders (generalized anxiety disorder, panic disorder, or social anxiety disorder, SAD; *n* = 67) compared to HCs (*n* = 65). These patients exhibited a brain‐PAD of +2.91 years after correction for antidepressant use (Han et al. 2021). Notably, this study did not examine individuals diagnosed with SPH. Few studies have examined abnormalities in brain structure in SPH participants. The largest study to date was recently performed by the ENIGMA‐Anxiety (Enhancing NeuroImaging Genetics through Meta‐Analysis) Working Group and demonstrated smaller subcortical volumes in the caudate nucleus, putamen, pallidum, and hippocampus, and larger thickness in the rostral middle frontal cortex, in SPH participants (*n* = 1456) compared to HCs (*n* = 2993; Hilbert et al. 2024), age 5–90 years. However, these correlates only manifested in a subgroup of SPH participants older than age 21 years. Participant age may moderate relations between anxiety disorders and structural brain alterations, as seen in an ENIGMA‐Anxiety mega‐analysis showing a negative diagnosis‐by‐age interaction for left putamen volume in adults with SAD (Groenewold et al. 2023). Age may therefore be expected to moderate associations with structural MRI‐based brain age in participants with anxiety disorders (Hilbert et al. 2024; Groenewold et al. 2023).

The ENIGMA‐Anxiety Working Group is a worldwide research collaboration which performs mega‐analyses on large, international multi‐site data (Bas‐Hoogendam et al. 2022). Given the high prevalence of SPH, its early onset, and its potential role as a marker for the development of additional psychopathology, the primary aim of the present ENIGMA‐Anxiety mega‐analysis is to compare brain age in a large multi‐site sample of adult SPH participants and HCs. We hypothesize that participants with SPH will have a greater brain‐PAD than HCs. Further, we examine associations between brain aging in SPH participants and symptom severity. We hypothesize that greater SPH symptom severity relates to a larger brain‐PAD.

## 2 Materials and Methods

### 2.1 Study Sample and Measures

This multi‐site collaborative study followed a mega‐analytic framework (Zugman et al. 2022) by including a subset of data (initially *n* = 693 SPH participants, *n* = 1824 HCs) from 17 research sites participating in the ENIGMA‐Anxiety Working Group (Hilbert et al. 2024; Bas‐Hoogendam et al. 2022), aged 22–75 years (Figure S1: age density plot). Demographic and clinical information appear in Table 1a and Table 1b. As in a prior report (Hilbert et al. 2024), SPH participants were excluded if they had a lifetime diagnosis of psychosis, schizophrenia, or bipolar disorder, but retained if they had other comorbidities. HCs were free of all current and past mental disorders.

**TABLE 1a:** Sociodemographic information for each study site included in the main analyses.

Site	Dx interview/questionnaire cut‐off	All			HC			SPH		
		*N*	% Female	Mean (SD) age	*N*	% Female	Mean (SD) age	*N*	% Female	Mean (SD) age
Barcelona	MINI	29	79.31	23.90 (2.06)	7	71.43	24.43 (2.44)	22	81.82	23.73 (1.96)
Dresden CRC940C5	CIDI	96	90.63	26.68 (5.31)	44	88.64	25.86 (5.07)	52	90.31	27.37 (5.47)
Dresden SPH subtypes	CIDI (*n* = 23 cut‐off, SNAQ, DFS)	57	75.44	25.74 (4.72)	18	66.67	25.11 (4.20)	39	79.49	26.03 (4.97)
FOR2107 MR	SCID	333	58.56	37.22 (12.55)	322	58.70	37.16 (12.55)	11	54.55	38.91 (12.98)
FOR2107 MS	SCID	170	66.47	32.58 (12.03)	146	65.75	31.66 (11.69)**	24	70.83	38.21 (12.86)**
Graz I	SCID	55	58.18	31.76 (10.18)	26	57.69	30.04 (8.67)	29	58.62	33.31 (11.30)
Graz II	Other	31	61.29	31.84 (11.24)	15	66.67	30.27 (10.05)	16	56.25	33.31 (12.20)
Greifswald spider snake	Cut‐off (SNAQ, SPQ)	20	100.00	25.00 (2.13)	10	100.00	25.80 (2.15)	10	100.00	24.20 (1.87)
Muenster dental phobia	SCID/DAS	26	73.08	30.19 (10.80)	14	64.29	28.71 (10.33)	12	83.33	31.92 (11.52)
Muenster SFBTRR‐58 C09	SCID	58	87.93	31.69 (9.63)	NA	NA	NA	58	87.93	31.69 (9.63)
Muenster spider	SCID	234	56.84	38.61 (11.16)	212	53.77*	39.85 (10.87)**	22	86.36*	26.64 (5.36)**
Protect‐AD	DSM‐5 SPH scale	34	58.82	37.15 (11.84)	NA	NA	NA	34	58.82	37.15 (11.84)
RepSpi	Cut‐off (SPQ)	19	84.21	24.16 (4.95)	11	72.73	22.73 (1.10)	8	100.00	26.13 (7.32)
SHIP	CIDI (*n* = 3 cut‐off)	438	60.05	53.95 (11.64)	303	51.82**	55.25 (12.02)**	135	78.52**	51.01 (10.17)**
Teneriffa	Other	32	71.88	35.72 (11.28)	6	50.00	25.83 (6.70)*	26	76.92	38.00 (10.91)*
Uppsala	Cut‐off (spider phobia questionnaire)	40	77.50	27.05 (7.66)	NA	NA	NA	40	77.50	27.05 (7.66)
Wuerzburg SFBTRR‐58 C09	SCID	62	85.48	30.45 (7.69)	NA	NA	NA	62	85.48	30.45 (7.69)
Total across samples	NA	1734	65.80	38.64 (14.55)	1134	58.82	40.42 (15.01)	600	79.00	35.28 (13.01)

*Note:* Table includes information for the final dataset after exclusions (e.g., due to FreeSurfer quality control failure). *p < 0.05; **p < 0.001 (independent samples *t*‐test and Chi‐square tests).

Abbreviations: CIDI, composite international diagnostic interview; Cut‐off, specific phobia participants included based on questionnaire cut‐off scores; DAS, dental anxiety scale; DFS, dental fear survey; Dresden CRC940C5, DFG Collaborative Research Centre 940, project C5; Dx, diagnosis; FOR2107 MR, DFG‐Research Group 2107 Marburg site; FOR2107 MS, DFG‐Research Group 2107 Muenster site; HC, healthy controls; MINI, mini international neuropsychiatric interview; Muenster SFBTRR‐58 C09, DFG Collaborative Research Centre Transregio 58, project C09, Muenster site; *N*, number; NA, not applicable; Protect‐AD, providing tools for effective care and treatment of anxiety disorders consortium, specific phobia sample; SCID, structured clinical interview for DSM; SD, standard deviation; SHIP: Study of Health in Pomerania; SNAQ, snake questionnaire; SPH, specific phobia; SPQ, spider questionnaire; Wuerzburg SFBTRR‐58 C09, DFG Collaborative Research Centre Transregio 58, project C09, Wuerzburg site.

**TABLE 1b:** SPH clinical information for each study site included in the main analyses.

Site	SPH	Comorbidity (lifetime/current)		Medication use		AOO	STAI‐T total scores	Symptom severity centiles	BDI II total scores
	% Current, lifetime or cut‐off	% ANX	% MDD	% any use	% SSRI/SNRI	Mean (SD)	Mean (SD)	Mean (SD)	Mean (SD)
Barcelona	100.00 current	0.00/0.00	0.00/0.00	NA	NA	NA	42.75 (9.66)	6.82 (1.40)	NA
Dresden CRC940C5	100.00 current	15.38/7.69	11.54 lifetime	0.00	0.00	5.73 (3.92)	35.56 (7.48)	7.85 (1.32)	4.04 (4.42)
Dresden SPH subtypes	41.03 current, 58.97 cut‐off	2.56/10.26	0.00/0.00	7.69	2.56 (*n* = 38 NA)	NA	NA	8.36 (0.54)	6.92 (8.12)
FOR2107 MR	100.00 current	18.18 current	9.09/72.73	63.64	45.45	NA	60.82 (6.72)	NA	24.82 (7.76)
FOR2107 MS	83.33 current, 16.67 lifetime	12.50/25.00	4.17/58.33	45.83	12.50 (*n* = 1 NA)	NA	52.79 (9.65)	NA	16.41 (7.47)
Graz I	100.00 current	0.00/0.00	0.00/0.00	0.00	0.00	13.42 (8.26)	NA	8.69 (1.49)	NA
Graz II	100.00 current	0.00/0.00	0.00/0.00	0.00	0.00	8.06 (4.81)	NA	8.50 (1.10)	NA
Greifswald spider snake	100.00 cut‐off	0.00/0.00	0.00/0.000	NA	NA	NA	NA	6.80 (1.23)	NA
Muenster dental phobia	100.00 cut‐off	0.00/0.00	0.00/0.00	0.00	NA	NA	35.75 (9.96)	7.42 (1.38)	5.09 (4.91)
Muenster SFBTRR‐58 C09	100.00 current	0.00/0.00	1.72/1.72	43.10	0.00	6.14 (5.18)	35.09 (8.24)	7.76 (0.68)	3.64 (4.40)
Muenster spider	100.00 current	0.00/0.00	0.00/0.00	0.00	0.00	NA	40.75 (9.99)	7.47 (1.65)	4.55 (3.71)
Protect‐AD	100.00 lifetime	73.53 lifetime	29.41 lifetime	0.00	NA	NA	NA	4.26 (2.06)	16.88 (10.93)
RepSpi	100.00 lifetime	0.00/0.00	0.00/0.00	0.00	NA	NA	35.63 (11.19)	7.50 (1.31)	NA
SHIP	0.74 current, 97.04 lifetime, 2.22 cut‐off	17.04 lifetime	28.15 lifetime	22.22 (*n* = 3 NA)	8.12	14.26 (11.34)	NA	NA	8.88 (9.22)
Teneriffa	100.00 current	0.00/0.00	0.00/0.00	0.00	0.00	NA	NA	7.31 (1.12)	NA
Uppsala	100.00 cut‐off	0.00/0.00	0.00/0.00	0.00	NA	5.74 (2.80)	NA	6.78 (1.03)	NA
Wuerzburg SFBTRR‐58 C09	100.00 current	0.00/0.00	1.61 lifetime	40.32	0.00	7.79 (3.83)	36.06 (9.27)	8.00 (0.81)	3.39 (4.28)
Total across all samples	55.83 current, 28.17 lifetime, 16.00 cut‐off	10.00/2.67	9.67/3.83	16.83	7.67 (*n* = 165 NA)	9.62 (8.48)	34.38 (9.14)	7.45 (1.61)	4.93 (6.29)

*Note:* Clinical information is not available for all participants within a site.

Abbreviations: ANX, anxiety; AOO, age of onset; BDI, beck depression inventory II; DFG‐Research Group 2107 Muenster site; Dresden CRC940C5, DFG Collaborative Research Centre 940, project C5; FOR2107 MR, DFG‐Research Group 2107 Marburg site; FOR2107 MS; MDD, major depressive disorder; Muenster SFBTRR‐58 C09, DFG Collaborative Research Centre Transregio 58, project C09, Muenster site; NA, not applicable; Protect‐AD, providing tools for effective care and treatment of anxiety disorders consortium, specific phobia sample; SD, standard deviation; SHIP, Study of Health in Pomerania; SNRI, serotonin and norepinephrine reuptake inhibitor; SPH, specific phobia; SSRI, selective serotonin reuptake inhibitor; STAI‐T, State‐Trait Anxiety Inventory‐Trait; Wuerzburg SFBTRR‐58 C09, DFG Collaborative Research Centre Transregio 58, project C09, Wuerzburg site.

This secondary data analysis received ethical clearance from the University of Cape Town Human Research Ethics Committee (reference: 675/2021). Participants provided written informed consent during participation with the original studies, and each research site obtained approval from their local ethics committees and institutional review boards to perform the original studies and to share the data with the ENIGMA‐Anxiety Working Group.

### 2.2 Image Acquisition and Processing

Research sites processed structural T1‐weighted magnetic resonance imaging (MRI) images using FreeSurfer (Fischl et al. 2002) and conducted quality control (QC) based on established ENIGMA protocols (https://enigma.ini.usc.edu/; Stein et al. 2012) to generate volumes for eight bilateral subcortical regions, surface area and cortical thickness for 34 bilateral regions and total intracranial volume, resulting in 77 features of brain structure in total. Cortical regions were parcellated according to the Desikan‐Killiany cortical atlas (Desikan et al. 2006). At the original research sites and central sites, investigators visually inspected the segmentations for failure and poor‐quality segmentation and generated summary statistics, outlier histograms, and boxplots. Next, subject‐level data was shared with central sites. We excluded participants if they had QC failure for > 10% of FreeSurfer regions across both hemispheres (SPH *n* = 82; HC *n* = 116). If participants had < 10% missing data, missing values for regions of interest due to QC failure were imputed with the median value of the specific region by the ENIGMA‐MDD brain age model. Data acquired for less than 10 participants per MRI scanner was excluded (*n* = 11 SPH participants from 3 scanners within Protect‐AD). Finally, HCs that were part of the sample used to train the ENIGMA‐MDD brain age model were excluded from the present study (*n* = 574).

The final sample consisted of 600 SPH participants (current *n* = 335; lifetime and not current *n* = 169; questionnaire‐based *n* = 96) and 1134 HCs from 17 research sites. SPH participants were considered ‘formally diagnosed’ if they were assessed with standardized clinical interviews, and ‘questionnaire‐based’ if recruited using questionnaire cut‐off scores, primarily used in university and community samples (Björkstrand et al. 2016; Feldker et al. 2017; Hilbert et al. 2015; Visser et al. 2016; Wendt et al. 2012).

### 2.3 Brain Age Calculations

We used a ridge regression brain age model developed by the ENIGMA‐MDD Working Group (https://photon‐ai.com/enigma_brainage). The model was developed separately in male (*n* = 12,353) and female (*n* = 14,182) HCs aged 18–75 years, using a mega‐analytic approach which uses random effects modeling on multi‐site, centralized data (Han et al. 2020). Specifically, normative models of the association between chronological age and 77 structural brain features were trained with the Python‐based *sklearn* package (Pedregosa et al. 2011) by combining left and right hemisphere FreeSurfer measures and calculating mean volumes of lateral ventricles and subcortical volumes and cortical thickness and surface area (Han et al. 2020). This model was validated in a test set of controls by calculating the absolute difference between brain age and chronological age in males (mean absolute error, MAE; standard deviation, SD) = 6.50 (4.91) and females = 6.84 (5.32). The Pearson correlation between chronological age and brain age was *r* = 0.85 (*p* < 0.001) in the male and *r* = 0.83 (*p* < 0.001) in the female model. The ENIGMA‐MDD brain age model was chosen for the present study as the model training sample and current study sample have similar age distribution, sex distribution, and geographical representation (primarily European; Han et al. 2020). Further, the model was developed within the ENIGMA Consortium; therefore, the same processing pipelines were followed, reducing methodological variability. In the current study, brain age was estimated for each participant by using their data for 77 brain structural features as input for the ENIGMA‐MDD model (for males or females, respectively).

### 2.4 Statistical Analysis

Statistics were conducted in SPSS version 29 and RStudio 4.2.2. Brain‐PAD was calculated by subtracting chronological age from estimated brain age for each participant. The fit of the ENIGMA‐MDD brain age model with the data was evaluated by calculating the MAE, Pearson correlation coefficients, and explained variance (*R* ^2^) between chronological age and brain age estimates in the whole sample, and separately in groups (SPH/HC; males/females). Further, model fit was assessed per research site.

The primary research question was tested using a linear mixed‐effects model (LME), as implemented in the *nlme* package, 4.2.2 in RStudio. Brain‐PAD was the outcome variable (unstandardised *β*‐values, measured in years), diagnosis (SPH/HC) was the predictor variable, scan site was included as random intercept (in all LMEs), and the mean‐centered chronological age, square of the mean‐centered chronological age (mean‐centered age^2^) and sex were included as covariates (in all LMEs). Diagnosis‐by‐sex and diagnosis‐by‐chronological‐age (both linear and quadratic) interaction terms were added to the model to evaluate whether the brain‐PAD is more apparent in specific age groups or in males or females. If these interactions did not significantly influence brain‐PAD, they were removed from the model to ensure optimal fit, based on the Akaike and Bayes information criterion (Akaike 1974; Schwarz 1978).

Before fitting between‐group LMEs, the dataset was checked for normality for age, brain‐PAD, MAE, and cortical thickness (Figures S2a–S5b). One SPH participant and one HC were identified as outliers due to extreme positive and negative brain‐PADs (Table S1 and Figure S6). Outliers were retained, and results were not impacted significantly by their exclusion (Table S2).

Certain research sites (FOR2107‐MS, Muenster Spider, SHIP, and Teneriffa, Table 1a) differed significantly between groups in mean age and proportion of female participants. Propensity score matching was conducted to match groups (SPH/HC) within these sites on age and sex using the *MatchIt* R package, version 4.5.0 (Ho et al. 2011). Nearest neighbor matching was implemented as it runs through all case participants and selects the closest eligible control participant for pairing (Thoemmes and Kim 2011). The difference between the propensity scores of each case and control unit was used as the distance measure, and a two HCs to one SPH participant ratio was chosen to optimize sample size. The LME was re‐run in this matched dataset.

We repeated the main LME in SPH participants with a formal current and lifetime SPH diagnosis only, excluding those classified as SPH solely based on questionnaire cut‐off scores (Table 1a). We repeated the analysis in formally diagnosed SPH participants with current SPH, as compared to HCs, excluding two research sites (Protect‐AD, RepSpi) which reported lifetime SPH only. Next, an exploratory analysis was run in the research sites with both SPH and HC participants, excluding those without HCs, to account for potential site effects.

To better delineate the effects of clinical variables on brain age, exploratory clinical subgroup analyses were run. Subgroup analyses were conducted comparing the brain‐PAD in unmedicated SPH participants and HCs, and as a supplementary analysis, in medicated SPH participants and HCs. Two comorbidity analyses were run, in which SPH participants with comorbid MDD were excluded, and with any comorbid anxiety disorder were excluded (generalized anxiety disorder, social anxiety disorder, panic disorder, agoraphobia, separation anxiety, and unspecified anxiety disorder). Of note, clinical information relating to medication use and comorbidities was not available for all research sites. False discovery rate (FDR) correction was applied to the *n* = 5 sub‐analyses of interest, specifically, formal current and lifetime SPH, current SPH, unmedicated SPH, no anxiety comorbidity, and no MDD comorbidity, with a significance threshold set at *p* < 0.05. Finally, a post hoc analysis was conducted where participants were split by median age of 35 years (22–35 years; 36–75 years), allowing for an approximately even sample size per age bin and maximum statistical power. The main LME was rerun in these age bins and repeated in formally diagnosed SPH and HCs within these age bins.

The second hypothesis, namely that greater symptom severity would predict greater brain‐PAD in SPH, was tested using an LME conducted in SPH participants only. Due to different symptom severity measures used across research sites, SPH participants were classified into severity centiles, in line with prior work on this sample (Hilbert et al. 2024; present study formally diagnosed SPH mean centile score = 7.49 [1.70]; questionnaire‐based mean centile score = 6.19 [2.72]). Brain‐PAD was the outcome variable, SPH symptom severity was the predictor variable, scanner site was included as a random intercept, and mean‐centered age, mean‐centered age^2^ and sex were included as covariates. This same model was run again with total State Trait Anxiety Inventory‐Trait (STAI‐Trait) scores (Spielberger and Vagg 1984) as the predictor variable. Analyses were repeated in formally diagnosed SPH participants (lifetime and current) only.

## 3 Results

### 3.1 Model Fit

The ENIGMA‐Anxiety SPH dataset fit well with the ENIGMA‐MDD brain age model, with comparable MAE (full sample = 7.26 [SD = 5.41], SPH = 7.43 [5.81], HC = 7.17 [5.19]), MAE weighted by sample age range (weighted MAE = 0.14 in full sample and per group), and Pearson correlations (full sample = 0.80, SPH = 0.76, HC = 0.81) between brain age and age to those observed in Han et al. (2020), using the same model (Tables S3–S5: model fit per study site).

### 3.2 Brain‐PAD in SPH Versus HCs

**FIGURE 1:**
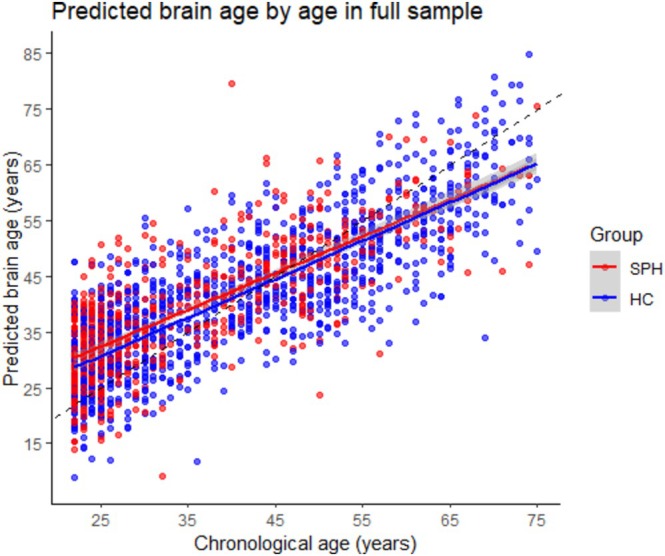
Chronological age plotted against predicted brain age in the full sample. HC, healthy controls; SPH, specific phobia. Diagonal dashed line represents the line of identity (*x* = *y*).

SPH participants (*n* = 600) had a mean chronological age of 35.28 (13.01), brain age of 39.18 (11.28) years, and thus mean brain‐PAD of 3.90 (8.59) years. HCs (*n* = 1134) had a mean chronological age of 40.42 (15.01), brain age of 41.33 (12.76) years and mean brain‐PAD of 0.91 (8.80) years. This brain‐PAD did not differ significantly between SPH participants and HCs, after adjusting for scanner site, mean‐centered age, mean‐centered age^2^ and sex (Cohen's *d* (95% confidence intervals) = 0.05 (−0.05–0.14), *p* = 0.32, Table 2; Figure S7: brain‐PAD residuals). A significant main effect of (mean‐centered) chronological age was observed (Table 2). After including an a priori diagnosis‐by‐age interaction term in the model, diagnosis was not significant (Cohen's *d* (95% confidence intervals) = 0.04 (−0.05–0.13), *p* = 0.39). However, we found a main effect of (mean‐centered) age, as well as an effect of mean‐centered age^2^ and a significant interaction of diagnosis‐by‐age (Table 2; Figure 1; Figure S8 shows scatter plots per site). Interactions with sex and diagnosis‐by‐age‐squared were not significant, and were therefore removed from the models.

**TABLE 2:** Between‐group differences (SPH vs. HC) in brain‐PAD, diagnosis‐by‐age interaction.

GLM without interaction term	*β* (SE)	*t*	*p*
Intercept (site)	1.89 (0.98)	1.93	0.05
Diagnosis	0.42 (0.43)	0.99	0.32
Sex	0.20 (0.37)	0.55	0.59
AgeC	−0.39 (0.02)	22.02	< 0.001
AgeC^2^	−1.54 × 10^−3^ (9.26 × 10^−4^)	−1.66	0.10

GLM with interaction term	*β* (SE)	*t*	*p*
Intercept (site)	1.83 (1.00)	1.83	0.07
Diagnosis	0.37 (0.43)	0.86	0.39
Sex	0.22 (0.37)	0.60	0.55
AgeC	−0.37 (0.02)	−19.18	< 0.001
AgeC^2^	−1.91 × 10^−3^ (9.37 × 10^−4^)	−2.04	0.04
Diagnosis‐by‐ageC	−0.07 (0.03)	−2.45	0.02

*Note:* β is measured in years.

Abbreviations: AgeC, mean‐centered chronological age; AgeC^2^, mean‐centered chronological age squared; Diagnosis‐ageC, diagnosis‐by‐age‐centered interaction term; GLM, general linear model; SE, standard error.

Subsequently, diagnosis‐by‐age‐squared and diagnosis‐by‐sex interaction terms were added to new models, one at a time. However, neither interaction term was significant (*β*_diagnosis‐by‐age_^2^ = −0.00 (0.00), *t*‐value = −0.80, *p* = 0.42; *β*_diagnosis‐by‐sex_ = −0.70 (0.83), *t*‐value = −0.85, *p* = 0.40), and both were removed to ensure optimal model fit (lower Akaike and Bayes information criterion: AIC = 11733.62, BIC = 11771.81).

An exploratory LME was run in research sites including both SPH (*n* = 480) and HC participants (*n* = 1134), to ensure the significant diagnosis‐by‐age interaction was not explained by site effects. Despite a smaller sample size, the diagnosis‐by‐age interaction remained a similar magnitude and approached statistical significance in this model (*β*_diagnosis‐by‐age_ = −0.06 (0.03), *p* = 0.06; Table S6), confirming that site effects do not explain the interaction. Finally, following nearest neighbor propensity score matching (Table S7, Figure S9); the brain‐PAD did not significantly differ between SPH participants and HCs, while the diagnosis‐by‐age interaction remained significant (Table S8).

### 3.3 Exploratory Clinical Subgroup Analyses

To assess if the diagnosis‐by‐age interaction was upheld in subgroups, post hoc sensitivity analyses were conducted. First, the primary LME was re‐run in formally diagnosed SPH participants compared to HCs (Table 3; Tables S9 and S10: clinical information). The results were consistent with those from the model using all SPH participants, with no significant difference in brain‐PAD between groups. Notably, the significant diagnosis‐by‐age interaction effect was larger than in the full sample and survived FDR correction. The interaction remained significant after FDR correction when the LME was conducted in current SPH only (without lifetime; Table 3). A subgroup analysis did not reveal an effect of diagnosis when comparing brain‐PAD between unmedicated SPH participants and HCs; however, the diagnosis‐by‐age interaction remained significant after FDR correction (Table 3). Two additional subgroup analyses were conducted in SPH participants without anxiety comorbidities (vs. HCs) and in SPH participants without MDD comorbidity (vs. HCs; Table 3). Brain‐PAD did not differ significantly between groups; however, the diagnosis‐by‐age interaction remained significant after FDR correction and therefore could not be explained by comorbid anxiety, comorbid MDD, or medication use.

**TABLE 3:** Between group differences in brain‐PAD, diagnosis and diagnosis‐by‐age interaction parameters.

	SPH	HC	Dx			Cohen's *d* (95% CI)	Dx‐by‐ageC			
	*n*	*n*	*β* (SE)	*t*	*p*		*β* (SE)	*t*	*p*	*pFDR*
SPH (no medication use) vs HCs	463	1134	0.03 (0.47)	0.07	0.94	0.00 (−0.09–0.10)	−0.07 (0.03)	−2.11	0.03	0.03
SPH (no ANX comorbidity) vs HCs	541	1134	0.18 (0.45)	0.40	0.69	0.02 (−0.08–0.11)	−0.06 (0.03)	−2.16	0.03	0.03
SPH (no MDD comorbidity) vs HCs	519	1134	0.43 (0.46)	0.93	0.35	0.04 (−0.05–0.14)	−0.07 (0.03)	−2.36	0.02	0.03
Diagnosed SPH (lifetime, current) vs HCs	504	1134	0.51 (0.44)	1.15	0.25	0.06 (−0.04–0.15)	−0.08 (0.03)	−2.85	0.00	0.02
Diagnosed SPH (current) vs HCs	335	1134	0.26 (0.64)	0.40	0.69	0.02 (−0.08–0.13)	−0.11 (0.05)	−2.41	0.02	0.03

*Note:* β is measured in years.

Abbreviations: ANX, anxiety; CI, confidence intervals; Dx, diagnosis; Dx‐by‐ageC, diagnosis‐by‐age‐centered interaction term; FDR, false discovery rate; HC, healthy controls; MDD, major depressive disorder; *n*, number; SE, standard error; SPH, specific phobia.

For completeness, supplementary analyses were conducted in medicated SPH versus HCs (Table S12), and in formally diagnosed SPH (lifetime, current) versus HCs, in only sites with both SPH and HC groups (Table S11; *β*_diagnosis_ = 0.49 [0.47], *p* = 0.30, Cohen's *d* [95% CI] = 0.06 [−0.05–0.16]; *β*_diagnosis‐by‐age_ = −0.07 [0.03], *p* = 0.02).

### 3.4 Post Hoc: Median Split Age

The consistently observed diagnosis‐by‐age interaction was explored further by running the primary LME in different age groups, split by median age (35 years) (Tables S13–S14: group clinical and demographic information; Figure S10a,b: brain age scatterplots). A difference in brain‐PAD of almost 1 year was observed between SPH participants and HCs in the younger age group; however, this was not significant (Table 4). No difference in brain‐PAD was observed between SPH participants or HCs in the older age group (Table 4). Due to the most pronounced diagnosis‐by‐age interaction effect found in formally diagnosed SPH participants (current and lifetime) compared to HCs, the post hoc analysis was repeated in this group. A significantly different brain‐PAD of +1.20 years was observed in SPH participants compared to HCs in the younger age group (*p* = 0.047), but not the older age group (Table 4).

**TABLE 4:** Between group differences in brain‐PAD, diagnosis parameters.

	SPH n	HC *n*	*β* (SE)	*t*	*p*	Cohen's *d* (95% CI)
All SPH vs HCs, 22–35 yrs	355	532	0.94 (0.57)	1.66	0.10	0.11 (−0.02–0.24)
All SPH vs HCs, 36–75 yrs	245	602	0.01 (0.64)	0.02	0.99	0.00 (−0.13–0.14)
Diagnosed SPH vs HCs, 22–35 yrs	272	532	1.20 (0.60)	1.99	0.05	0.14 (0.00–0.28)
Diagnosed SPH vs HCs, 36–75 yrs	232	602	0.07 (0.65)	0.11	0.91	0.01 (−0.13–0.14)

*Note:* β is measured in years.

Abbreviations: CI, confidence intervals; HC, healthy controls; *n*, number; SE, standard error; SPH, specific phobia; vs., versus; yrs., years.

### 3.5 Symptom Severity Analyses

SPH symptom severity centile score and STAI‐T score were not significantly associated with brain‐PAD in SPH participants (symptom severity centile score: *n* = 427, *β*_symptom‐severity_ = 0.18 (0.24), *t*‐value = 0.74, *p* = 0.46; STAI‐T score: *n* = 266, *β*_STAI‐T_ = 0.02 (0.04), *t*‐value = 0.41, *p* = 0.68). When conducting analyses in formally diagnosed SPH participants only, *β*‐values were slightly larger but still non‐significant (symptom severity centile score: *n* = 334, *β*_symptom‐severity_ = 0.19 (0.26), *t*‐value = 0.72, *p* = 0.47; STAI‐T score: *n* = 246, *β*_STAI‐T_ = 0.03 (0.04), *t*‐value = 0.58, *p* = 0.56).

## 4 Discussion

This multi‐site mega‐analysis examined whether brain‐PAD differed between adult SPH participants and HCs. Brain‐PAD did not relate to SPH in the full sample, or when limiting the analyses to SPH participants with a formal diagnosis (versus. HCs). However, a significant diagnosis‐by‐age interaction persisted across sensitivity analyses, remained after the removal of outliers (Table S2), and was most evident in formally diagnosed SPH participants versus HCs. For the purposes of interpretation, exploratory post hoc analyses were conducted, splitting participants by median age. This showed a subtle advanced brain‐PAD in younger formally diagnosed SPH participants compared to HCs of +1.20 (0.60) years, Cohen's *d* = 0.14 (age: 22–35 years), but not in older participants (age: 36–75 years). No associations were identified between brain‐PAD and symptom severity scores in SPH participants. The diagnosis‐by‐age interaction suggests a slightly earlier end of maturational processes, or an advanced early decline in neurostructural measures.

### 4.1 Brain Aging Patterns According to Age

The brain‐PAD is correlated with chronological age. Brain age models may underestimate age in older samples and overestimate age in younger samples, particularly when participant age deviates from the training sample mean age (Ballester et al. 2022; Butler et al. 2021). Including age (linear and quadratic) in the LMEs can correct for this age bias as effectively as applying correction to the brain‐PAD metric (de Lange et al. 2022; de Lange and Cole 2020). Still, brain age prediction accuracy may be impacted by differing age ranges of the training and test samples (Han et al. 2020; Cole et al. 2019). Here, the model training and study samples had a similar age distribution. Further, the model demonstrated reasonable fit with the present dataset (Han et al. 2020). After adjusting for age in analyses, a greater effect may still be expected in older individuals, due to accelerated aging during older age (Ballester et al. 2022). However, post hoc analyses showed a positive brain‐PAD in younger formally diagnosed SPH participants compared to controls, but not in older participants, suggesting accentuated structural brain aging in young adults with SPH.

Biological and lifestyle mechanisms may impact biological aging. Biological factors, such as oxidative stress, contribute to premature cell death and have been associated with both biological aging and anxiety disorders (Bouayed et al. 2009; Tsaluchidu et al. 2008), but these may be more relevant in older individuals. Maturation of brain regions such as the amygdala, hippocampus and prefrontal cortex in youth may be vulnerable to unhealthy lifestyle factors, including chronic stress (Giedd and Rapoport 2010; Romeo 2017), such as that experienced in SPH (Romeo 2013). Given evidence of ongoing brain maturation throughout the first three decades of life (Arain et al. 2013; Hedman et al. 2012; Frangou et al. 2022), the diagnosis‐by‐age interaction may be indicative of a slightly earlier end of brain maturation or a subtle ‘advanced’ early decline in structural brain measures. To better understand this process, longitudinal brain age work is necessary.

The size of the brain‐PAD observed in the younger formally diagnosed SPH group is smaller than observed previously in a multi‐site study of adult clinically diagnosed anxiety disorder participants (+2.91 years after correcting for antidepressant use; Han et al. 2021). However, Han et al. (2021) did not assess SPH participants. Further, the group difference in brain‐PAD is similar in size to MDD (approximately +1 year; Han et al. 2020; Han et al. 2022), and smaller than bipolar disorder and psychotic disorders (+2 years and + 3 years respectively; Blake et al. 2023; Ballester et al. 2022). The small effect identified in the present study is in line with research showing that structural brain differences in anxiety disorders tend to be subtle (Groenewold et al. 2023; Goodkind et al. 2015). Nevertheless, a recent mega‐analysis in a larger version of the present sample suggests that SPH structural effect sizes may exceed that reported in other anxiety disorders (Hilbert et al. 2024).

### 4.2 Clinical Subgroups and Symptom Severity

Greater brain‐PAD was observed in younger formally diagnosed SPH, but not in the full age range of formally diagnosed SPH. The formally diagnosed SPH subgroup may present with more clinically severe and persistent disorder than questionnaire‐based participants, who were primarily recruited from community and university settings. The questionnaires did not assess significant impairment or disorder persistence (e.g., minimum duration of 6 months), whereas clinical interviews include these criteria (American Psychiatric Association 2022; Oliveira et al. 2014; Zsido et al. 2018). Further, formally diagnosed SPH had a slightly higher mean symptom severity centile score than questionnaire‐based SPH. Therefore, it may be expected that advanced brain aging is most pronounced in formally diagnosed participants, as was the case in our younger subgroup.

Research has suggested a potential subtle neuroprotective effect of antidepressant use against brain aging (Han et al. 2021). The present study found no significant difference in brain‐PAD between either medicated or unmedicated SPH and controls. However, only a relatively small percentage of included SPH participants used selective serotonin reuptake inhibitors (SSRIs) or serotonin and norepinephrine reuptake inhibitors (Table 1a), possibly due to limited evidence that SSRIs are beneficial in SPH (Singh and Singh 2016). Potential increased disorder severity in participants taking psychotropic medications could offset possible neuroprotective effects. This could offer one explanation for the trend in our sub‐analysis of medicated SPH participants compared to HCs, where a brain‐PAD of +1.10 years was observed (Table S12), although this did not reach significance between groups.

SPH patients commonly have comorbid psychopathologies, which may become more severe than the phobia (Wardenaar et al. 2017; Sanderson et al. 1990), highlighting the importance of examining whether comorbidities impact the diagnosis‐by‐age interaction. We found no significant differences between SPH participants without anxiety disorder comorbidities and HCs, or between SPH participants without MDD comorbidity and HCs. The diagnosis‐by‐age interaction term remained in both models, suggesting that the age‐specific relationship between diagnosis and brain‐PAD may not be explained by comorbid anxiety and MDD diagnoses. However, information related to comorbidity was not available for all research sites. Descriptively, younger participants showed lower frequencies of anxiety and depressive comorbidities, and medication use, than older participants, although no formal statistical comparisons were conducted.

Previous studies have found a higher brain‐PAD with increased symptom severity, primarily in psychotic disorders (Koutsouleris et al. 2014; Lieslehto et al. 2021; Schnack et al. 2016). Further, one study detected an association between higher brain‐PAD and higher anxiety and depressive symptoms across individuals diagnosed with anxiety disorders, MDD and HCs (Han et al. 2021). The present study did not detect an association between brain‐PAD and SPH symptom severity centile scores or STAI‐T scores (measuring trait anxiety; Spielberger and Vagg 1984) in the SPH group. The lack of significant relationships between clinical symptoms and brain structure aligns with the finding of a recent ENIGMA‐Anxiety study, from which the present study sample was derived (Hilbert et al. 2024). The lack of findings suggests that a relationship between SPH symptom severity and brain aging is not present, however, heterogeneity in clinical measures and smaller sample sizes (compared to the primary analysis), may have resulted in decreased sensitivity to detect associations.

### 4.3 Study Limitations and Strengths

This is the first mega‐analysis to investigate brain aging in a large, international sample of SPH participants. Multisite collaborations, such as ENIGMA, have the major benefit of large sample sizes which increase statistical power to detect group differences (Bas‐Hoogendam et al. 2022; Thompson et al. 2020). Further, MRI data pre‐processing and quality checking was harmonized across research sites, enhancing methodological homogeneity. Finally, the brain age model was trained on a large independent sample and fit well with the present dataset, demonstrating similar fit statistics to Han et al. (2020); Han et al. (2022).

Some limitations of the present study deserve to be mentioned. While considered a well‐validated measure (Spielberger and Vagg 1984), some research suggests that the STAI‐T may not specifically measure trait anxiety, but instead a more general negative affect (Knowles and Olatunji 2020). Clinical information was not prospectively harmonized across research sites and not available for all sites, resulting in smaller sample sizes in sub‐analyses and decreased power to detect group differences and associations. We attempted to mitigate site heterogeneity in diagnostic assessments through sensitivity analyses, though some variability may persist. In addition, not all research sites had control participants. However, supplementary analyses demonstrated that the diagnosis‐by‐age finding was not impacted by site‐effects.

Furthermore, while the present brain age model is sensitive to the effects of aging due to the inclusion of cortical thickness measures (Wang et al. 2014), it predicts age based on the whole brain. Certain models can estimate brain age for specific brain regions, providing additional information on regional brain aging patterns (Kaufmann et al. 2019). Further, brain age models with MAEs may be more sensitive to detecting group‐level case–control differences, rather than individual‐level predictions (Schulz et al. 2025). Additionally, caution is important when interpreting findings relating to advanced brain aging or maturation, as underlying mechanisms remain to be elucidated (Whitmore et al. 2023). Finally, anxiety disorders commonly start early in life (Kessler et al. 2012), therefore, brain age studies in children and adolescents with anxiety disorders could provide clarity regarding brain maturation and the direction of the relationship between brain aging and anxiety disorders.

## 5 Conclusion

This ENIGMA‐Anxiety mega‐analysis did not identify significantly advanced brain aging in the full sample of SPH participants. However, a diagnosis‐by‐age interaction was present across analyses, and was particularly evident in formally diagnosed SPH participants compared to HCs. Further, a subtle advanced brain aging was identified in post hoc analyses in formally diagnosed young adults with SPH. This finding potentially indicates a slightly earlier end to maturational processes or an advanced decline in structural brain measures. The findings in the present study suggest that advanced brain aging may be more apparent in formally diagnosed participants, indicating the importance of clinical severity, impairment and persistence. Future studies should consider cross‐disorder designs, including multiple anxiety disorders. In addition, research is needed in younger participants, on regional brain age patterns in SPH, and using longitudinal designs to provide clarity on which developmental or aging processes may be implicated by brain age models.

## Funding

This work was supported by NIH Big Data to Knowledge (BD2K) (U54 EB020403), Oppenheimer Memorial Trust (22148/01), Boehringer Ingelheim Fonds, NWO, Rubicon (019.201SG.022), Medical Delta, Dutch Research Agenda, NWA/NeurolabNL (NWA.1418.22.025), Interdisciplinary Center for Clinical Research (IZKF) of the medical faculty of Münster (Dan3/016/26), Swedish Research Council, Swedish Brain Foundation, Bundesministerium für Bildung und Forschung. German Research Foundation (DFG), MICIU/AEI/10 and ESF+, Weijerhost Foundation, NIMH Intramural Research Award, Carnegie Corporation of New York, German Federal Ministry of Education and Research, Ministry of Cultural Affairs, Social Ministry of the Federal State of Mecklenburg‐West Pomerania, and Siemens Healthcare.

## Disclosure

H.J.G. has received travel grants and speakers honoraria from Neuraxpharm, Servier, Indorsia and Janssen Cilag. K.H. is a scientific advisor to the Aury Care GmbH, which develops an AI‐based chatbot providing mental health support. He holds virtual stock options in Aury Care GmbH. Previously, this relationship was with the Mental Tech GmbH, from which the Aury Care GmbH took over product development. All other authors report no biomedical financial interests or potential conflicts of interest.

## Supporting information


**Figure S1:** Age density plot per diagnostic group.
**Figure S2a–S2b:** Age boxplots.
**Figure S3a–S3b:** Brain‐PAD boxplots.
**Figure S4a–S4b:** MAE boxplots.
**Figure S5a–S5b:** Cortical thickness boxplots.
**Table S1:** Brain‐PAD extreme values.
**Figure S6:** Boxplot of brain‐PAD extreme values.
**Table S2:** Between‐group differences (SPH *n* = 599 vs. HC *n* = 1133) in brain‐PAD (outlier removal).
**Table S3:** Model fit statistics, full sample, and per diagnostic group and sex.
**Table S4:** Mean absolute error per study site and diagnostic group.
**Table S5:** Pearson's *R* and *R* ^2^: brain age and brain‐PAD and age, per study site and diagnostic group.
**Figure S7:** Brain‐PAD residuals plot for primary linear mixed‐effects model without interaction term.
**Figure S8:** Brain age by age scatterplots per diagnostic group and research site.
**Table S6:** Between‐group differences (SPH *n* = 480 vs. HC *n* = 1134) in brain‐PAD, sites with HCs.
**Table S7:** Summary of balance for matched and unmatched data per study site.
**Figure S9:** Density plots for age and sex (0 = male; 1 = female), per matched research site.
**Table S8:** Between‐group differences (SPH *n* = 577 vs. HC *n* = 766) in brain‐PAD, matched dataset.
**Table S9:** Clinical information for formally diagnosed SPH participants (*n* = 504).
**Table S10:** Clinical information for questionnaire cut‐off SPH participants.
**Table S11:** Between‐group differences in brain‐PAD (formally diagnosed SPH, lifetime and current *n* = 384 vs. HCs *n* = 1134) in sites with controls versus HCs.
**Table S12:** Between‐group differences in brain‐PAD (medicated SPH *n* = 101 vs. HC *n* = 1134).
**Table S13:** Demographic and clinical information, participants ≤ 35.
**Table S14:** Demographic and clinical information, participants > 35.
**Figure S10a:** Scatterplot of brain age by age in younger formally diagnosed SPH and HCs.
**Figure S10b:** Scatterplot of brain age by age in older formally diagnosed SPH and HCs.

## Data Availability

The ENIGMA‐Anxiety Working Group is open to sharing the data and code from this investigation to researchers for secondary data analysis. To request access to brain morphometric, clinical, and demographic data, an analysis plan can be submitted to the ENIGMA‐Anxiety Working Group (http://enigma.ini.usc.edu/ongoing/enigma‐anxiety/). All data access is contingent on approval by PIs from contributing samples. The results of this project were presented in the form of a poster presentation at Society of Biological Psychiatry in Austin, Texas, on 09 May 2024, and at the Southern African Neuroscience Society in Durban, South Africa, on 19 July 2024. The current manuscript is substantively different from the previous work, as it is a full archival report, rather than a poster presentation or an abstract. In addition, a preprint of the article is available on medRxiv, with doi:https://doi.org/10.1101/2025.03.19.25323474. Blake, K. V., Hilbert, K., Ipser, J. C., Han, L. K., Bas‐Hoogendam, J. M., Aghajani, M., … & Groenewold, N. A. (2024). 67. An Enigma‐Anxiety Mega‐Analysis Investigating Brain Ageing in Specific Phobia. Biological Psychiatry, 95 (10), S126‐S127.
